# From Environment to Genome and Back: A Lesson from *HFE* Mutations

**DOI:** 10.3390/ijms21103505

**Published:** 2020-05-15

**Authors:** Raffaela Rametta, Marica Meroni, Paola Dongiovanni

**Affiliations:** 1 General Medicine and Metabolic Diseases, Fondazione IRCCS Ca’ Granda Ospedale Maggiore Policlinico, Pad. Granelli, via F Sforza 35, 20122 Milan, Italy; raffaela.rametta@policlinico.mi.it (R.R.); maricameroni11@gmail.com (M.M.); 2 Department of Pathophysiology and Transplantation, Università degli Studi di Milano, 20122 Milan, Italy

**Keywords:** Hereditary hemochromatosis, HFE, iron metabolism, polyphenols, vitamins, miRNAs, insulin signaling

## Abstract

The environment and the human genome are closely entangled and many genetic variations that occur in human populations are the result of adaptive selection to ancestral environmental (mainly dietary) conditions. However, the selected mutations may become maladaptive when environmental conditions change, thus becoming candidates for diseases. Hereditary hemochromatosis (HH) is a potentially lethal disease leading to iron accumulation mostly due to mutations in the *HFE* gene. Indeed, homozygosity for the C282Y *HFE* mutation is associated with the primary iron overload phenotype. However, both penetrance of the C282Y variant and the clinical manifestation of the disease are extremely variable, suggesting that other genetic, epigenetic and environmental factors play a role in the development of HH, as well as, and in its progression to end-stage liver diseases. Alcohol consumption and dietary habits may impact on the phenotypic expression of *HFE*-related hemochromatosis. Indeed, dietary components and bioactive molecules can affect iron status both directly by modulating its absorption during digestion and indirectly by the epigenetic modification of genes involved in its uptake, storage and recycling. Thus, the premise of this review is to discuss how environmental pressures led to the selection of *HFE* mutations and whether nutritional and lifestyle interventions may exert beneficial effects on HH outcomes and comorbidities.

## 1. Introduction

Humans are able to modify the surrounding environment by exploiting progressively advanced technologies, with the purpose to derive water, nourishment, energy and shelter. This process is defined as “niche construction” and may be profoundly influenced by behavioral and cultural traits [1]. Unlike other organisms, humans are able to carry or adapt the constructed niche in a new environment. An example of a culturally-induced niche is represented by the introduction of agriculture and the shift from hunting to the domestication of animals, which led to modifications in the diet composition during the Neolithic era that spread throughout Europe from east to west and south to north about 6000 years ago (4000 BC) [2,3].

The human genome may be affected by environmental changes and inherited variations are in part the result of adaptive evolution to ancient environmental conditions. Casually occurring single nucleotide polymorphisms (SNPs) represent the primary example of genetic variations and can impact on the organism’s response to the fluctuant nutrient supply, providing an adaptive advantage to the environment. Thus, favorable mutations have been selected and expanded within ancient populations [4,5], which explains their high incidence in the modern ones. However, such favorable genetic variants may become a modern-day candidate risk factor for several disorders, due to changes in environmental conditions. The present review aims to explore how environmental pressures lead to adaptive alterations of the genome through the selection of favorable mutations and whether it is possible to introduce dietary and lifestyle interventions in the management of iron-related hepatic disorders caused by selected inherited variations that have become maladaptive in the modern age.

## 2. From the Environment to Genome: When Diet and Lifestyle May Change Our Genes

Hereditary hemochromatosis (HH) represents one of the most important examples of diseases induced by maladaptive mutations that have been selected during evolutionary processes. It is a common autosomal recessive disorder characterized by excessive iron deposition in the liver and extrahepatic tissues due to inappropriately high iron absorption. Early symptoms of HH may include weakness, lethargy, arthralgias and impotence. Later manifestations include cirrhosis, hepatocellular cancer (HCC), osteoporosis, cardiomyopathy, dysrhythmia, diabetes (T2D) and hypogonadism. Currently, phlebotomy represents the main therapeutic option for HH treatment [6].

Hepcidin (HAMP) is the hepatic hormone that regulates iron absorption. It controls systemic iron availability though the negative regulation of its target ferroportin (FPN), a transmembrane iron efflux channel highly expressed in enterocytes, macrophages and hepatocytes. Hepcidin regulators are activated by positive *stimuli* (iron status and inflammation), and negative ones (erythropoietic activity and hypoxia). Mutations occurring in genes involved in hepcidin expression and regulation, which cause a defective production or activity of the hormone, lead to HH [7,8].

Most cases of HH are attributable to mutations in the *HFE* gene, which encodes a non-classical MHC class 1 protein involved in the downregulation of iron absorption [9]. HFE, which is predominantly located at the hepatocyte cell surface, competes with transferrin (Tf) to bind transferrin receptor-1 (TfR1) thus reducing TfR1–Tf interactions and negatively regulating iron uptake. With increasing plasma iron levels, the iron-loaded Tf (holo-Tf) gains high affinity for TfR1, HFE is displaced from the HFE-TfR1 complex and becomes available to associate with transferrin receptor-2 (TfR2) and hemojuvelin (HJV). The formed HFE–TfR2–HJV complex triggers the bone morphogenetic protein (BMP)/SMAD signaling pathway to hepcidin gene (HAMP) expression [10].

HFE is also expressed in intestinal enterocytes. Indeed, HFE was found as a complex with TfR1 on the basolateral membrane of enterocytes in the duodenal crypt cells, as well as, in the villus enterocytes of the small intestine. It has been speculated that HFE may act as a plasma iron sensor by modulating Tf-mediated uptake or the release of dietary iron by these cells [11].

The transition c.845G > A (rs1800562), resulting in the amino acid substitution from cysteine to tyrosine at position 282 (C282Y) in the HFE protein, blocks the ability of HFE to downregulate iron absorption by preventing its expression on the cell surface. C282Y homozygosity is associated with iron primary overload phenotype in more than 60% of Europeans [12]. The transversion c.187C > G (rs1799945), which leads to the histidine-to-aspartic acid substitution at position 63 (H63D), has a milder effect on iron absorption. Only 5%–7% of HH patients are compound heterozygotes for C282Y/H63D, whereas heterozygosity for C282Y alone or homozygosity for H63D, is rarely encountered in HH [13]. Lastly, the c.193A > T (rs1800730) transversion, which causes the serine to cysteine substitution at position 65 (S65C) in HFE protein, has been involved in the development of a less severe form of hemochromatosis only in combination with C282Y or H63D mutations [14,15].

The C282Y variant is predominantly enriched in the European population and it is less frequent (Hispanics and Pacific Islanders) or nearly absent (Asians and Africans) in non-Caucasian populations [16]. In addition, the prevalence of C282Y homozygosity follows a strong geographical distribution among Europeans with the highest frequencies in Northern Europe (10.9% in Ireland, 9.7% in Scotland, 8.2% in Wales, 7.8% in Brittany, 7.3% in Norway and 7% in Denmark) and the lowest frequencies in southern Europe (ranging from 1 to 5% in the Mediterranean area) [17].

Evolutionary analyses suggest that the C282Y mutation in the *HFE* gene may have originated from a mutation in a single Celtic or Viking ancestor around 4000 BC [18] and was spread in central Europe following the migratory flows. Distante and colleagues suggested that the spread of the C282Y variant could be the result of an adaptation to a dietary shift from a hunter-gatherer diet based on wild foods (rich in iron) to a Neolithic diet based on cereal and dairy food (poor in iron) [19].

Indeed, before the Neolithic Age, the subsistence strategies were based on wild flora and fauna (game, fish, shellfish, insects, nuts, roots and vegetables). Red meat and many species of shellfish provided a rich source of highly digestible heme-iron [20]. During the Neolithic Age, the introduction of agriculture (mainly of cereal grains and other seeds) and animal breeding caused a very drastic change in eating habits leading to the primary dependence on cereals and dairy products, thus shifting human subsistence toward a high carbohydrate/low iron diet [2]. The combination of grains and bovine milk product consumption reduced effective iron absorption in Neolithic farmers. Indeed, on one hand, phytates localized on the surface of cereal grains and other seeds can chelate dietary iron and several other minerals, making them physiologically unavailable [21,22]. On the other hand, the calcium-rich bovine milk is iron-free as it lacks in lactoferrin and thus it may impair the absorption of non-heme iron [23,24].

In addition, the sedentary lifestyle imposed by agriculture resulted in increased fertility and rapid population growth with a consequent inter-birth interval reduction, which makes it difficult for women to restore body iron stores between two pregnancies [25,26].

As a consequence of changes in lifestyle and food habits, Neolithic farmers were exposed to the risk of iron deficiency anemia. Hence a C282Y mutation in *HFE*, leading to increased iron absorption, provides a clear selective advantage and represents the genome adaptive response to the environmental changes [27].

**Figure 1:**
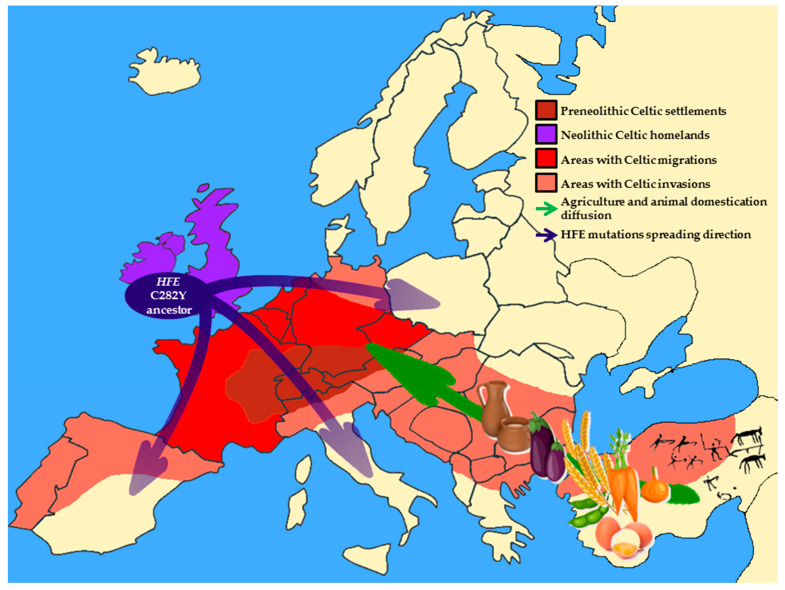
Schematic representation of Europe during the Neolithic Age. Brown area indicates pre-Neolithic original Celtic settlements; purple area shows Celtic homeland during the Neolithic Age; red area is regions occupied by the main Celtic settlements after Neolithic migrations; orange areas indicate the areas reached by the Celtic invasions. Blue arrows show the gradient of *HFE* C282Y spreading throughout Europe. The green arrow represents the diffusion of agriculture and the domestication of animals.

The interaction between Celtic culture diffusion and Neolithic niche spreading throughout Europe, which resulted in the selection of *HFE* C282Y mutation during the Neolithic Age is schematically represented in Figure 1.

## 3. Going Back to Genome: How to Change the Environment to Treat the Disease

Epidemiological studies suggested that the penetrance of the commonest C282Y *HFE* mutation on iron overload phenotype is extremely variable ranging from 2% to 38% in males and from 1% to 10% in females. Furthermore, there is huge inter-individual variability in the clinical manifestations of HH. Indeed, homozygous individuals may be asymptomatic or they can have a more complicated disease showing hemochromatosis-associated comorbidities (fibrosis or cirrhosis) or other clinically relevant manifestations (T2D, cardiovascular diseases, arthropathy and hypogonadism) [28,29,30]. This evidence supports the notion that other genetic, epigenetic and environmental factors play a role in the development of the disease and in its progression to end-stage liver diseases. Moreover, several environmental and behavioral factors such as diet composition and alcohol consumption may impact on phenotypic expression of *HFE*-related hemochromatosis.

### 3.1. Alcohol Consumption

Alcohol abuse is one of the major non-genetic modifiers of iron accumulation in HH patients and may contribute to the progression of symptoms [31]. Indeed, it has been described that excessive alcohol intake precipitates cirrhosis onset in patients with *HFE*-related hemochromatosis [32]. Moreover, studies conducted in 224 C282Y homozygous patients showed that heavy drinkers (with an alcoholic intake > 60 g dayly) had higher iron indices [31,33] and a nine-fold increased risk of developing cirrhosis than those consuming safer alcohol doses [31,34,35]. However, the lack of large perspective epidemiological studies reporting the accurate history of alcohol consumption, does not allow for the assessment of the absolute risk of its intake, regardless of the amount ingested, on the HH phenotype.

Nevertheless, in healthy subjects, alcohol consumption reduced hepcidin expression and increased dietary iron absorption, leading to increased serum iron parameters [36,37,38]. In addition, in vitro and in vivo studies suggest a direct role of alcohol in the downregulation of HAMP expression in hepatoma cells and in livers of both short-term alcohol-exposed mice [36] and chronic alcohol-treated rats fed with iron-enriched diets [39,40].

### 3.2. Dietary Iron Sources: Heme and Non-Heme Dietary Iron

Iron bioavailability and its dietary intake are determinants of iron status in the general population [41,42]. Dietary iron exists as heme and non-heme bound forms, which are absorbed by intestinal epithelial cells through different mechanisms. While heme-iron is taken up intact and then released from the porphyrin ring by heme oxygenase, non-heme iron requires active transport by divalent metal transporter 1 (DMT1) to cross the enterocyte membrane [43,44]. This difference makes heme-iron more readily available than the non-heme one [45]. Indeed, it has been estimated that heme-iron, derived from foods of animal source, accounts for 10%–15% of total iron intake in meat-eating populations. Conversely, the bioavailability of non-heme iron is lower and is closely related to the balance between inhibitors and enhancers of absorption, which are present in foods and in the body iron storages [45].

Nevertheless, the potential diet-related effects on iron accumulation in HH are poorly investigated. C282Y homozygous patients showed increased absorption of both heme- and non-heme iron [46]. Moreover, in the same patients the percentage of non-heme iron absorption from the un-supplemented meal was inversely related to the body iron stores, suggesting that its regulation is impaired in these patients. On the contrary, heme-iron absorption seems to be unaffected by body iron stores [46]. However, when meals were enriched with iron and modified to improve its availability, an exaggerated absorptive response to non-heme iron independently of iron stores was observed in patients’ heterozygous first relatives [46]. These observations emphasize the impact that the introduction of iron-supplemented foods could have in subjects at risk of developing significant iron overload.

In a recent systematic review [47], Moretti and colleagues estimated whether and to what extent dietary iron restriction and modulation of dietary iron bioavailability in both C282Y homozygous and idiopathic patients could represent a therapeutic approach in the prevention and management of HH. These authors highlighted that iron bioavailability is 2- to 10-fold higher in patients with clinically overt disease compared to wild-type [48] and it is influenced by the food matrix [49]. However, scant direct evidence is available to support the hypothesis that dietary modulation alone can reduce iron accumulation in HH subjects. Indeed, the results obtained from small cross-sectional studies [50,51,52,53] remain still controversial because of the limited number of subjects included and the potential confounding effects of chronic subclinical inflammation that impact on iron status. Nevertheless, they suggest that a dietary strategy aimed at reducing iron consumption and bioavailability, paralleled by an adequate intake of other essential nutrients, may provide an auxiliary measure to inhibit iron accumulation and reduce the number of required phlebotomy procedures in HH patients [47].

### 3.3. Insulin Resistance and Iron Homeostasis

Several lines of evidence indicate that ferritin levels and body iron stores should be considered as hallmarks of metabolic syndrome (MetS) whose key pathogenic feature is represented by insulin resistance (IR) [54,55,56]. Patients affected by nonalcoholic fatty liver disease (NAFLD), which is considered the hepatic manifestation of MetS [57], are frequently characterized by mild hepatic iron accumulation [58]. The pathological condition that encompasses fatty liver, increased ferritin levels and enhanced body iron depots in the presence of IR, is referred to as dysmetabolic iron overload syndrome (DIOS). DIOS is observed in 15%–30% of patients with MetS and it is considered the most common iron overload disorder [59].

Excessive iron storages catalyze reactive oxygen species (ROS) overproduction and in turn, lipid peroxidation [60]. In keeping with this data, it has been demonstrated that high-fat diet (HFD) administration induces hepatic iron deposition, paralleled by ferritin induction and by *TfR-1* and iron regulated protein-1 (*IRP1*) overexpression in rodents [61].

Likewise, patients affected by HH often display reduced insulin sensitivity and release, thus increasing their susceptibility to developing NAFLD [58]. In particular, depending on the magnitude of iron depots, around 53%–80% of HH patients develop T2D [62]. In a cross-sectional population-based study, Tuomainen and colleagues revealed a statistically significant alteration of glucose homeostasis in 1013 middle-aged men with hyperferritinemia, in eastern Finland [63]. Moreover, in a case-control study, the risk of developing T2D in subjects with elevated iron stores was attested at 2.4-fold higher than controls [64]. The correlation between serum ferritin levels and T2D has been further confirmed even in 9486 U.S. adults aged > or = 20 years from the Third National Health and Nutrition Examination Survey (NHANES; 1988–1994) [65] and in 27,548 individuals recruited in the European Prospective Investigation of Cancer (EPIC)-Norfolk Cohort Study [66].

The prevalence of the C282Y *HFE* mutation is significantly increased in patients with NAFLD compared to controls and the difference is more striking in patients with hyperferritinemia than in those without [58]. Furthermore, it has been demonstrated that iron deposition in hepatocytes and in non-parenchymal cells is associated with more severe liver damage in 587 Italian patients with NAFLD carrying the *HFE* variations [67]. These patients develop NAFLD even in the presence of less severe metabolic abnormalities, suggesting that iron overload may contribute to NAFLD onset and progression towards end-stage liver diseases [67].

Insulin signaling and iron homeostasis are closely entangled, as revealed by a broad number of clinical and preclinical studies [68,69]. Insulin is able to stimulate ferritin synthesis and facilitates iron uptake. In turn, increased iron concentrations lead to peripheral hyperinsulinemia [70]. The likely mechanism through which the iron excess impairs hepatocyte insulin sensitivity is due to its direct interfering with the insulin receptor and intracellular insulin signaling cascade [71]. Moreover, pancreatic β-cells are extremely sensitive to iron-related ROS, thus precipitating T2D and its comorbidities onset [60].

According to this notion, iron depletion through phlebotomy along with lifestyle modifications ameliorate insulin sensitivity and secretion, reduces glycaemia and improves liver function in NAFLD patients carrying the C282Y allele [72,73,74,75]. Moreover, iron depletion is essential in the cellular response to hypoxic conditions, stabilizing the hypoxia-inducible factor (HIF)-1α, which is also involved in glucose uptake by inducing glucose transporter 1 (Glut1) and anaerobic glucose metabolism [76,77]. Dongiovanni and colleagues widely investigated the impact of iron depletion by deferoxamine on insulin signaling and glucose uptake in HepG2 cells and in rat livers. These authors have demonstrated that deferoxamine-induced iron depletion stabilizes *HIF-1α* and induces glucose uptake through *Glut1*, glucose utilization and insulin sensitivity [76].

Conversely, in C57Bl/6 wild-type male mice, dietary iron supplementation resulted in hypogonadism with a significant reduction of serum levels of testosterone [78], hyperglycemia, hyperinsulinemia, IR and hepatic iron accumulation. As a consequence of iron accumulation in adipocytes, iron-supplemented mice display lower adipose tissue mass and peripheral IR, testified by decreased phospho-Akt/Akt ratio and overexpression of adipokines including cytokine signaling-3 (Socs3), a target of hepcidin involved in IR [59].

Intriguingly, our unpublished data demonstrate that Hfe heterozygous (Hfe+/–) mice have a similar glucose concentration compared to wild-type (Hfe+/+) when they are fed a standard diet (94.8 ± 11 vs. 114 ± 41 mg/dL, respectively). However, after the administration of an iron-enriched diet for eight weeks, glucose levels were increased in both genotypes, but more so in the wild-type (157.8 ± 16 vs. 184.6 ± 27 mg/dL; *p* < 0.05). Moreover, fasting glucose levels and IR after insulin tolerance test (ITT) were higher in the wild-type compared to Hfe+/– mice treated with the same diet. Consistently, in wild-type mice, IR was paralleled by an impairment in insulin signaling activation and by a lower hepatic phospho-Akt/Akt ratio.

As we expected, hepatic iron and splenic contents were more severely increased in Hfe+/– mice compared to their wild-type littermates (700 ± 35 vs. 300 ± 15 μg/100 mg dry hepatic tissue and 450 ± 25 vs. 150 ± 10 μg/100 mg dry spleen tissue; *p* < 0.001), after the administration of the iron-enriched diet. However, the iron concentration in visceral adipose tissue was significantly enhanced in wild-type mice compared to Hfe+/– mice (10 ± 3 vs. 30 ± 5 μg/100 mg dry adipose tissue; *p* = 0.03), suggesting that adipose tissue iron overload may play a strong causative role in IR onset, more so than hepatic iron accumulation. Consistently, Hfe+/– mice fed an iron-enriched diet showed a decrease in adipose tissue resistin levels and an increase in adiponectin gene expression compared to their wild-type siblings fed the same diet, confirming that wild-type mice develop stronger peripheral IR than Hfe+/–. This effect seems to be mediated by the persistence of hepcidin presence in wild-type mice even after an iron challenge, as it occurs in DIOS patients differently from HH [79,80]. Indeed, DIOS and NAFLD disorders show elevated hepcidin levels related to hyperferritinemia. To confirm this hypothesis, we have recently demonstrated that iron challenge in iron-depleted DIOS patients did not restrain iron absorption despite adequate hepcidin production, suggesting that hepcidin resistance (i.e., the impaired hepcidin activity) and not the deficit of hormone production is involved in DIOS pathogenesis [81].

These novel findings pave the way to explore therapeutic strategies and clinical trials, based on hepcidin concentration control in order to limit iron overload in patients. Moreover, nutritional personalized therapeutic approaches aimed at reducing iron, carbohydrate and saturated fatty acid intake from diet may slow the raising of T2D and hepatic complications in HH patients.

### 3.4. Role of Bioactive Compounds in Iron Metabolism

Bioactive compounds are extra-nutritional substances naturally present in small quantities in foods that impact on metabolism at several levels. Indeed, they can influence intestinal transit, modify nutrient absorption and excretion and exert detoxifying and antioxidant actions [82].

Bioactive compounds affect dietary iron absorption by acting as inhibitors or enhancers of non-heme iron uptake [83]. Moreover, many bioactive compounds are phytochelators, i.e., plant products that have detoxifying properties and are able to prevent iron oxidative damage. Therefore, they can be successfully used (together with synthetic iron chelator drugs) in both primary iron overload disease, such as HH, and in secondary iron overload disease, namely β-Thalassemia [84].

The manipulation of diet composition should take into account the impact of these molecules on iron bioavailability to tailor a dietetic approach in subjects with perturbations of iron homeostasis. Indeed, due to their chelating and redox properties, some of these compounds reduce serum or tissue iron levels thereby their consumption should be recommended in patients with iron overload and avoided in those with iron deficiency. Moreover, bioactive compounds promoting iron mobilization or increasing iron uptake, which are beneficial for patients with iron deficiency, should be avoided in iron overloaded patients.

The next sections summarize the role of bioactive compounds in iron metabolism, focusing on their possible use in HH management and treatment. The main dietary bioactive compounds and their impact on iron metabolism are listed in Table 1.

#### 3.4.1. Dietary Inhibitors of Iron Absorption: Phytates, Polyphenols, Calcium and Milk Soybean and Egg Proteins

##### Phytates

Phytic acid (myo-inositol-6-phosphate) is the main inhibitor of iron absorption. Phytates are largely present in all edible plant seeds (grains, legumes, oilseed and nuts) and to a lesser extent in roots, tubers and vegetables [85]. Due to their negative charge, in physiological conditions, phytates chelate multivalent cations including iron, zinc, magnesium and calcium [86]. The resulting complexes are soluble in the acidic environment of the stomach but precipitate in the small intestine where the pH reaches the neutral values, making iron (and other minerals) poorly absorbable. The phytate content in foods was found to be inversely related to the iron absorption [87]. Moreover, phytates exert their negative effects on iron absorption in a dose-dependent manner starting at very low concentrations (2–10 mg/meal) [88,89]. In a mouse model of iron overload, the chelating action of phytates attenuates iron-induced oxidative stress and alleviates liver injury [90]. Therefore, due to the iron lowering effect of these nutraceutical compounds, the introduction of nutrients rich in phytates could be considered a possible dietary intervention aimed to reduce iron bioavailability in HH patients who are predisposed to liver damage.

##### Polyphenols

Polyphenols are plant secondary metabolites, which contain one or more aromatic rings bearing one or more hydroxyl groups. They exert a pivotal role in the interactions between plants and the surrounding environment, as well as in plant reproduction and defense. Indeed, phenols are needed for the attraction of pollinators or seed-dispersing animals, for the protection against herbivores, microbe and viruses, ultraviolet radiation, oxidant agents and fluctuation of nutrients in soil [91]. Polyphenols are present in a wide variety of vegetarian food (vegetables, fruit, some cereals and legumes) and beverages (tea, coffee and wine). The phenolic compounds during digestion bind iron, likely through their galloyl groups, and polymerize it in insoluble complexes, which make iron unavailable for absorption [92].

In the past 40 years, iron-chelating properties of polyphenols have been explored [93,94] in several studies investigating their effects on iron absorption in humans. The consumption of yod kratin (a highly polyphenols-rich vegetable) reduced iron absorption from a composite meal by almost 90% in 83 Thai male healthy volunteers [95]. Polyphenols-rich beverages such as tea, coffee and cacao have been shown to lower iron absorption accordingly to the total quantity of polyphenols content [96,97,98,99].

Interestingly, the consumption of polyphenols-containing beverages has been suggested as a useful strategy to reduce iron absorption in iron overload disorders. Indeed, Kaltwasser and colleagues demonstrated that iron absorption was significantly reduced when a meal was accompanied by tea instead of water in 18 HH patients. Moreover, regular tea drinking with meals reduced the frequency of phlebotomies required in the management of the diseases [100].

It has been shown that food supplementation with polyphenolic extract from tea and rosemary reduced iron absorption in 27 premenopausal women with normal iron stores, suggesting that increased flavonoids supplementations may maintain a relatively low iron status and may, therefore, reduce the risk of iron overload [101].

Among polyphenols, flavonoids (primarily quercetin) are considered the main dietary inhibitors of duodenal iron absorption. However, the exact molecular mechanisms through which flavonoids reduce iron bioavailability remain elusive. The majority of the evidence comes from studies on quercetin effects on non-heme iron absorption in vivo. It has been demonstrated in a rat model that quercetin chelates iron in the intestinal lumen and blocks it inside the enterocytes, by increasing iron apical uptake and preventing basolateral transport into the bloodstream [102], thus reducing intestinal iron absorption. Indeed, long-term administration of 50 mg/kg/daily for 10 days of quercetin by gavages decreases duodenal *DMT1*, *duodenal cytochrome b (Dcytb)* and *Fpn* mRNA levels and leads to animal iron depletion. Moreover, intraperitoneal short-term administration of 50 mg/kg of quercetin resulted in a significant reduction of iron parameters and upregulation of HAMP expression in the duodenum [103]. This evidence is intriguing and has a potential therapeutic translational relevance for patients with iron deficiency as well as for those with iron overload. Indeed, quercetin supplementation was successfully tested in 84 patients with β-Thalassemia, who usually develop secondary iron overload due to blood transfusion therapy. Administration of 500 mg/daily of quercetin after a meal significantly reduced serum iron levels, ferritin, transferrin saturation and inflammatory markers such as tumor necrosis factor-α (TNF-α) and high-sensitivity C-reactive protein) compared to both baseline and placebo groups suggesting that quercetin may reduce iron overload in beta-thalassemic patients by exerting its iron-chelating activity [104].

However, quercetin effects on iron overloaded patients have not been investigated yet. Nevertheless, other polyphenols that belong to the flavonoid family were tested with conflicting results. Preliminary data suggest that consumption of sylibin, another flavonoid derived from milk thistle, reduced postprandial iron serum levels in 10 C282Y homozygous patients [105]. Conversely, in vitro studies demonstrated that the iron-chelating ability of grape seed proanthocyanidin extract, a flavonoid of the tannin class, was equivalent to 30 mM of the iron chelator deferoxamine, in a 50 mM solution of ferrous sulfate [106]. In a double-blind randomized controlled study, the nutritional supplementation with procyanidins in HH patients did not significantly reduce dietary iron absorption during an iron-rich meal [107].

These conflicting results are possibly attributable to the fact that the effectiveness of polyphenols in chelating iron could be impaired by their intrinsic bioavailability, by the incorrect experimental timing, or by the different methods used to detect iron absorption. Therefore, further studies on a larger set of patients are required to clarify the role of polyphenols in iron absorption.

Nevertheless, polyphenols provide many health benefits and the consumption of polyphenol enriched foods is widespread. Consistently, we could speculate that chronic consumption of foods scarce in iron and rich in inhibitors of iron availability, such as in a vegan or vegetarian diet, might contribute to the diffusion of iron deficiency syndromes among determinate groups of individuals such as children, pregnant women or the elderly. Conversely, consumption of a polyphenol-enriched diet might be beneficial for individuals at risk of iron overload, such as HH patients.

##### Calcium

Calcium is the only dietary factor able to inhibit both heme- and non-heme iron absorption [108] in a dose-dependent fashion [109]. The mechanism underlying its inhibitory effect is not fully understood but it has been proposed that calcium may reduce the initial iron mucosal uptake by enterocytes without affecting its basolateral transfer [110]. Moreover, calcium has been shown to delay iron uptake in the intestinal epithelial cells in animal models [111]. In addition, an in vitro study suggested that calcium reduces iron bioavailability by decreasing DMT1 expression at the apical cell membrane, thereby downregulating iron transport into the cell [112].

The question of whether the individual iron status may play a role in iron-calcium interaction has to be clearly addressed. Cook and colleagues described a significant inhibitory effect of calcium administered as calcium carbonate in 61 volunteers with low iron stores [108], the same treatment inhibited iron absorption in postmenopausal women with normal iron stores [113]. A recent review [114] of epidemiological studies that focused on the relationship between iron status and calcium intake indicates that calcium supplementation is unlikely to have significant effects on iron stores, although iron supplementation may be indicated during pregnancy to prevent anemia in women with high milk intake [115].

##### Proteins

Proteins deriving from soybean, milk and eggs have been shown to inhibit iron absorption [116]. Indeed, casein and whey, the two major bovine milk fractions, and egg white seem to prevent iron absorption in humans [117,118]. Furthermore, an epidemiological study of the phenotypical expression of *HFE* C282Y, H63D and S65C mutations in 1294 Danish men demonstrated that high milk consumption decreased the clinical phenotypic expression of C282Y and H63D mutations by lowering serum ferritin levels [119].

Among proteins of a plant source, soybean derived ones have strong inhibitory effects on iron absorption [89]. However, when they were enzymatically digested by hydrolases to remove phytates from the matrix, the percentage of iron absorption increased by 19-fold suggesting that the iron absorption preventing the effect of soy is mediated by both phytates and proteins [120].

#### 3.4.2. Dietary Enhancer of Iron Absorption: Vitamins and “Meat Factor”

##### Vitamins

Vitamins are essential micronutrients required for metabolism maintenance as they are cofactors for several enzymes. Vitamin A is a fat-soluble antioxidant molecule and it is also involved in iron homeostasis. It has been demonstrated that Vitamin A positively affects intestinal iron absorption. Indeed, its supplementation has been shown to prevent the inhibitory effect of phytates and polyphenols on iron absorption by sequestering iron in soluble complexes thus improving non-heme iron absorption from phytates-rich foods such as rice, wheat and corn [121].

Consistently, Vitamin A serum levels are positively related to iron indices (serum iron, hemoglobin and transferrin saturation) in humans [122] and its deficiency often co-occurs with iron deficiency anemia in some populations [123,124]. Nevertheless, Vitamin A deficiency has been reported also in patients with idiopathic hemochromatosis [125]. Interestingly, this finding was confirmed in a more recent case-control study [126]. Indeed, C282Y homozygous patients showed lower Vitamin A serum levels than healthy controls at baseline. Remarkably, the reduction of oxidative stress and the mild anemic status induced by venesection therapy restored Vitamin A serum levels close to those of healthy subjects.

Moreover, Vitamin A deficiency has been associated with reduced iron incorporation in erythrocytes [127], alteration in red blood cell morphology [128] and mild anemia [129] but also with increased iron absorption and accumulation in the liver and spleen [130] in animal models.

The role of Vitamin A in the regulation of iron homeostasis is intricate and may involve several mechanisms. Experimental evidence showed that vitamin A deficiency is positively related to the upregulation of hepatic *HAMP* expression in vivo without affecting the expression of genes involved in iron absorption. Moreover, it induces FPN expression in vitro in a hepcidin-independent manner, thus suggesting that vitamin A deficiency promotes iron absorption by inducing its mobilization [131]. In addition, the exposure of Caco-2, an enterocyte cell line, to the Vitamin A precursor β-carotene reduced intracellular ferritin and in turn increased FPN expression leading to the release of intracellular trapped iron [132].

Vitamin C or ascorbic acid is the main enhancer of non-heme iron absorption. Vitamin C promotes iron absorption through different synergic mechanisms: (1) improving gastric iron solubility by acidifying the stomach content; (2) preventing the formation of ferric insoluble complexes by reducing iron from ferric to ferrous form thus making it available for transport by DMT1; (3) maintaining iron solubility in the duodenal neutral environment by chelating ferrous iron [133].

Several single-meal studies in human volunteers have shown that Vitamin C improves iron absorption both as a food source [134,135,136,137] and as a supplement [136,138]. Noteworthy, the positive effect of Vitamin C on iron absorption widely varies according to the timing of intake, the amount ingested and the meal composition. Indeed, increased iron absorption was not observed when Vitamin C was administered several hours before an iron-content meal [139]. In addition, 50 mg of Vitamin C increased iron absorption to 61% from a hamburger meal and to 164% from a pizza meal [136]. Meal composition, as well as the whole food matrix, impact the effectiveness of Vitamin C in modulating iron absorption. Although Vitamin C has been shown to overcome the negative effects of the inhibitors of iron absorption, including phytates [88], polyphenols [140], calcium and milk proteins [141], the high content in polyphenols and phytates of some fruits and vegetables may abolish the positive effect of Vitamin C [142,143].

While the potentiating effects of vitamin C on iron absorption in single-meal studies have been convincingly demonstrated, the impact of vitamin C on iron status is not unknown. Limited available evidence deriving from long-term studies in subjects with low body iron stores are conflicting. Indeed, two studies showed a significant improvement of iron status in pre-school children with mainly plant-based diets [144,145] and in young women with low iron stores who consumed 164 mg/day of Vitamin C for 16 weeks [146]. Conversely, Vitamin C supplementation (1500 mg/day for 5 weeks or 50 mg/day for 24 weeks) failed to improve serum ferritin in women with low body iron stores consuming western diets [147,148].

However, further studies are required to assess the effectiveness of Vitamin C supplementation in improving iron status as differences in sample size, length of intervention, time and dose of supplementation and age of participants make these results difficult to define.

The impact of Vitamin C on iron stores in HH patients has not been investigated yet. However, Milward and colleagues showed retrospectively that non-citrus fruit consumption had significant protective effects on iron stores in HH male patients, independently of *HFE* genotype [53]. Although neither citrus fruit consumption nor Vitamin C supplementation increased iron stores, the consumption of 14 or more pieces of non-citrus fruit per week resulted in a 20% reduction of ferritin serum levels, suggesting that a diet rich in non-citrus fruits should be recommended in HH patients whose iron levels are not well controlled by phlebotomy or blood donations. Thus, it could represent a helpful strategy to reduce the frequency of phlebotomy required to reach iron depletion in HH patients.

##### Muscle Tissues from Meat, Fish and Poultry: The “Meat Factor” Effect

An animal-based diet has an important role in iron balance not only for its own iron content but also for the ability of animal foodstuffs (mainly meat, fish and poultry) to enhance non-heme iron absorption [149]. Indeed, in their systematic review, Jackson and colleagues suggested a positive correlation between the intake of animal flesh foods and increased iron stores in healthy adults within developed countries [150]. In addition, several single-meal studies showed that muscle tissues from meat, fish and poultry have a positive effect on iron absorption from vegetarian meals [151], to the same extent as Vitamin C [152]. Indeed, addition of chicken beef or fish to maize meal increased iron absorption by 2–3 fold [153]. To date, the nature of the “meat factor” remains elusive and the mechanisms underlying the enhancing effect of muscle tissues are still debated. Zang and colleagues suggested that muscle tissues, defined as a “meat factor”, may enhance non-heme iron absorption by promoting iron solubility in the stomach (where it stimulates gastric acid secretion) and by maintaining it (both by stimulating gastrin and/or other gastric factors and by chelating non-heme iron) during digestion in neutral pH conditions of the duodenum thus making non-heme iron readily absorbable [154]. More recent evidence suggests that the “meat factor” enhancing effect on iron absorption could be attributable to the ability to reduce and chelate iron of Cysteine-containing peptides derived from myofibrillar protein digestion, glycosaminoglycans and L-α-glycerophosphocholine [155,156,157].

#### 3.4.3. Prevention of Detrimental Effects of Iron-Induced Oxidative Stress: Antioxidants

##### Vitamin E

Vitamin E (RRR-alpha-tocopherol) is an important lipid-soluble antioxidant compound. It acts as a scavenger of free radicals protecting biological membranes from radical-mediated injury [158,159]. The liver is involved in Vitamin E metabolism. Indeed, after duodenal uptake, Vitamin E is mainly transferred as chylomicrons to hepatic parenchymal cells where it is re-oxidized and secreted with very low-density lipoprotein (VLDL) into the bloodstream [160].

Lipid peroxidation, due to iron-mediated oxidative stress, plays a pivotal role in the pathogenesis of liver damage in iron overload conditions [161]. Indeed, it results in a loss of integrity and impaired function of cellular organelles in experimental iron overload models [162,163,164]. Interestingly, lipid peroxidation was increased and antioxidant potential was impaired in HH patients [165].

Vitamin E supplementation exerts a protective effect on redox damage both in vivo and in vitro [166,167]. Conversely, Vitamin E deficiency increases lipid peroxidation and promotes oxidant damage in hepatocytes [168,169]. Moreover, iron overload has been shown to reduce Vitamin E levels in animal models [170]. Consistently, patients with HH have lower Vitamin E serum levels than healthy controls [171,172]. Interestingly, in HH patients who have reached iron depletion by phlebotomy, serum levels of Vitamin E were comparable to those of controls, suggesting that the impairment of antioxidant potential due to Vitamin E deficiency may have a role in the pathogenesis of HH [172]. Therefore, it is reasonable to assume that Vitamin E supplementation in these patients may have beneficial effects on iron overload-induced redox injury by preventing tissue damage.

The positive effects of Vitamin E supplementation in preventing lipid peroxidation induced by iron free-radicals were successfully confirmed in animal models of dietary iron overload whereas they remain to be deeper investigated in HH patients [173,174].

##### Phenolic Antioxidants: Flavonoids, Ferulic acid and Resveratrol

Among polyphenols, Flavonoids represent the major class of antioxidants. A flavonoid-rich extract of citrus fruits (orange and bergamot) has been shown to chelate iron and to reduce ROS production and membrane lipid oxidation by activating catalase enzymes in lung epithelial cell lines [175].

Similarly, grape seed extract (GSE) and epigallocatechin-3-gallate (EGCG), a tea-derived catechin, have potent antioxidant properties. Indeed, EGCG prevents oxidative stress by activating nuclear factor erythroid 2 related factor 2 (*Nrf2*) [176], a master transcriptional regulator of antioxidant genes [177]. Moreover, EGCG prevents intestinal iron absorption by reducing basolateral iron export in Caco-2 cells [178] and it may improve iron status and restore the redox potential in patients with HH. GSE-derived anthocyanins have been shown to antagonize oxidative injury by increasing total antioxidant potential and decreasing iron content through downregulation of Hamp and upregulation of Fpn expression in human embryo-derived hepatic cells [179]. Curcumin decreases iron levels in bone marrow, the spleen and the liver. In a mouse model of diet-induced subclinical iron deficiency, curcumin administration resulted in a dose-dependent reduction of hematocrit, hemoglobin, serum iron and transferrin saturation, exacerbating iron deficiency anemia. Consistently with the iron depletion status of these animals, curcumin treatment induced the activation of hepatic *IRP* and *TfR-1* as well as the reduction of hepatic ferritin and *HAMP*. In vitro in HepG2 cells, curcumin treatment reduced *HAMP* expression and induced ferroportin thus leading to iron efflux from cells [180].

Ferulic acid is a phenolic compound abundantly present in grains, artichokes, coffee and fruits. Its antioxidant effect is mediated by the neutralization of free radicals [181]. Iron overloaded mice treated with sodium ferulate showed reduced hepatic iron-induced oxidative stress, which led to improving the mitochondrial membrane potential, reversing mitochondrial swelling and decreasing the production of ROS, therefore, reducing liver injury [182]. However, the effects of ferulic acid on iron-induced oxidative stress have not yet been investigated in HH patients.

Resveratrol (3,5,4-trans-trihydroxystilbene) is a compound that belongs to the stilbene subclass of polyphenols. Resveratrol has pleiotropic effects and acts as an antioxidant, anti-inflammatory and anti-apoptotic [183]. In murine models of genetic HH exposed to chronic iron-overload, resveratrol reduced iron-induced hepatosplenomegaly, oxidative stress, hepatic fibrosis and inflammation and a pro-apoptotic status. Resveratrol supplementation provided protection from iron-mediated tissue injury in these models, whereas it did not affect the degree of hepatic iron overload. Indeed, by restoring sirtuin 1 (*SIRT1*) expression, resveratrol induced forkhead box O1 (FOXO1) deacetylation and activated FOXO1-dependent anti-oxidant response during oxidative stress [184]. Therefore, resveratrol supplementation may represent a potential therapy for iron-induced liver damage in patients with HH.

### 3.5. Epigenetic Modifiers of HFE: miRNAs-Nutrients Interaction in the Modulation of HH Phenotype

Epigenetic modifiers in HH can represent novel molecular predictors that can determine not only the early risk assessment but also the disease progression and prognosis, providing a new perspective on HH management. Indeed, the phenotypic discrepancies of HH manifestations may also be the consequence of the complex gene–environment interplay, explained by epigenetic mechanisms, hereditable but reversible modifications that modulate the transcriptome in response to environmental cues, without altering DNA sequence [185]. Epigenetics embraces a broad number of events such as alterations of DNA nucleotides (i.e., methylation of CpG dinucleotides, known as CpG islands), modifications of histones and regulation of transcription by altering mRNA stability through small RNA molecules such as microRNAs (miRNAs). Specifically, miRNAs are short non-protein coding, single-strand RNAs of 19–22 nucleotides that regulate gene expression and cell-to-cell communications as it occurs between hepatocytes and Kupffer cells or hepatic stellate cells (HSCs) during hepatic inflammation and fibrogenesis [185]. Indeed, miRNAs can target mRNAs through complementary base-pairing, thereby leading to the post-transcriptional repression of targeted protein-coding genes [186,187,188].

Dietary habits may strongly affect the hepatic epigenetic landscape, introducing, for example, modifications of cellular homeostasis and macro/micronutrient metabolism thus influencing the health status but also affect pathological conditions [189,190]. More than two thousand miRNAs have been described [191]. Evidence has revealed that they are encoded in a wide variety of intronic, exonic or intergenic sequences and that they directly target up to 60% of all human genes [191]. In particular, miRNAs may exert either the silencing of the target mRNAs or the repression of protein synthesis through different mechanisms [186]. Therefore, miRNAs may regulate at multiple levels iron metabolism by targeting the majority of genes involved in iron uptake, storage and recycling at cellular and systemic level [192]. Likewise, each miRNA may target multiple genes (multi-functionality) or multiple miRNAs can target a single gene (redundancy), supporting the notion that miRNAs may have an extensive regulatory ability in iron homeostasis and a strikingly diverse effect on health status and disease [186]. In particular, heme plays a crucial regulatory role in miRNA biogenesis, through the direct interaction with DiGeorge syndrome critical region 8 (DGCR8), which participates in precursor-miRNA cleavages [193]. As a consequence, during heme deficiency, the maturation of miRNA precursors is predicted to be slow, resulting in a global decrease of mature miRNA abundance.

Moreover, several miRNAs among which miR-22, miR-200a, and miR-320 are predicted to bind the 3′UTR sequence of *TfR-1*, essential for iron uptake from the blood. Specifically, the overexpression of miR-320 hampers TfR-1 expression and subsequently lowers iron availability and cell proliferation [194]. Nevertheless, miR-15a/b, miR-223 and Let-7d are involved in the transferrin cycle, interfering with the iron release from the endosome via DMT1. The iron retention in endosomes may indicate functional cellular iron deficiency [192]. Lactoferrin, which is another member of transferrin family, along with its receptor has been characterized as a functional target of miR-214 in both HC11 and MCF7 cancer cells [195]. The overexpression of miR-214 markedly decreased lactoferrin expression, also reducing apoptotic processes [195]. The post-transcriptional expression of the lactoferrin receptor, mainly localized in the apical membrane of enterocytes, is mediated also by miR-584 in both Caco-2 cells and in the mouse small intestine during the perinatal period, thus participating in the absorption of lactoferrin-bound iron from breast milk [196]. Finally, miR-200b has been well known to regulate iron cellular storage mediating ferritin regulation [197].

An example of miRNAs that are well established to be closely entangled with iron metabolism is represented by miR-122. Indeed, miR-122, the most abundant miRNA in the liver, is reduced in genetically-induced murine models of HH and accordingly even in hepatic biopsies of C282Y homozygous patients. Consistently, the inhibition of miR-122 in wild-type mice through intraperitoneal injection of antagomiR, hesitates in hampered circulating and tissue iron concentrations in spleen and liver, mildly impairing hematopoiesis [198]. Moreover, miR-122 deficiency enhances the expression of Hfe, hemojuvelin (Hjv) and Hamp, which are involved in the sensing of systemic iron levels [198]. miRNA-target prediction analyses, indeed, reveal the presence of miR-122 binding sites on *Hfe, Hjv* and *Hamp* 3′ UTR regulatory sequences. These observations have been confirmed in primary hepatocytes, after the transfection of miR-122 mimics or inhibitors, further corroborating that miR-122 is enabled to modulate systemic iron utilization and may be implicated in the prevention of iron overload and in iron overload-related complications, such as cirrhosis and hepatocellular carcinoma [192].

Among the bioactive compounds, several polyphenols i.e., quercetin, coffee polyphenols and grape seed proanthocyanidin may directly influence miR-122 expression, affecting, in turn, different metabolic pathways such as de novo lipogenesis, ameliorating body fat deposition and preventing diet-induced hepatic abnormalities in murine models [199,200]. The modulation of iron absorption by polyphenol-related miRNAs has been more clearly investigated [102]. Lesjak M. and collaborators demonstrate that quercetin, the most abundant dietary polyphenol and potent iron chelator, is able to downregulate Fpn levels both in in vivo and in vitro models. Indeed, the downregulation of *Fpn* has been attributable to miR-17–3p and its interaction with *Fpn* 3′UTR sequence. This data sustains the idea that dietary polyphenols severely impact on iron bioavailability thereby limiting the rate of intestinal iron absorption [102].

However, the field of diet-epigenome interaction in HH and its comorbidities remains largely unexplored. Overall, this evidence supports the hypothesis that a polyphenol-supplemented dietary approach to HH patients may improve tissue iron accumulation and eventually ameliorate liver injuries induced by iron depots, whereby modulating miRNA expression. This concept may provide a new example of gene-environment interaction that should be deeply investigated in the future.

**Figure 2:**
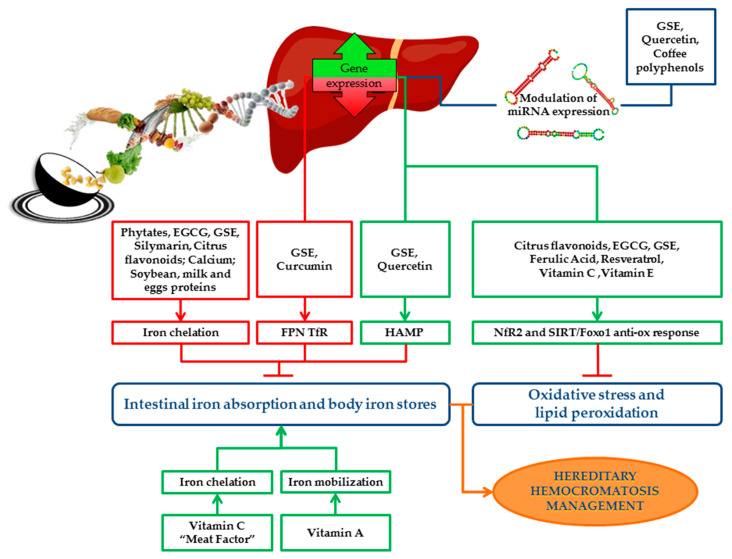
Schematic representation of the impact of bioactive compounds and dietary factors on iron metabolism. Dietary factors and bioactive compounds regulate iron metabolism directly, by enhancing/inhibiting its absorption, storage and recycling, or indirectly by the modulation of miRNAs, which regulate the expression of iron genes. Bioactive compounds act as antioxidants and protect cells and tissues from detrimental effects of iron overload by reducing oxidative stress and lipid peroxidation. The picture shows the main pathways induced (green boxes) or downregulated (red boxes) by bioactive compounds and the final effect on iron absorption, body iron stores, oxidative stress and lipid peroxidation (green arrows indicate positive effects; red “T” arrows indicate inhibitory ones). Nutritional interventions aimed at reducing iron absorption and improving iron-induced oxidative stress may be useful and safe strategies supporting phlebotomy in the management and treatment of HH patients. EGCG: Epigallocatechin-3-gallate; GSE: Grape seed extract; FPN: Ferroportin; TfR: Transferrin receptor; HAMP: Hepcidin antimicrobial peptide gene; ROS: Reactive oxygen species; Nrf2: Nuclear factor erythroid 2 related factor 2; SIRT1: Sirtuin 1; FOXO1: Forkhead box O1.

An overview of the impact of bioactive compounds and dietary factors on iron metabolism is schematically represented in Figure 2.

## 4. Concluding Remarks

Following in the footsteps of *HFE* mutations in this fascinating journey from the environment to the genome and back, we learned that environment and genome are strictly intertwined and they communicate with each other through dietary habits and lifestyle. Indeed, the agricultural revolution which occurred during the Neolithic era has profoundly changed eating habits shifting them from an iron-rich/low-carbohydrate diet to a low-iron/high-carbohydrate diet, thus exposing people to a high risk of anemia. In this context, the casually occurred *HFE* mutations, which favor iron accumulation, were selected as a genome response to environmental stress.

Most recently, the new revolution in dietary habits leading to the diffusion of the Western diet makes *HFE* mutations the most important risk factors for iron overload in Caucasian people of Northern European descent, thus explaining the evolutionary origin of the most common inherited metabolic disease in Western Countries.

The primary treatment to remove iron excess in HH patients is phlebotomy. It is also suggested to limit dietary iron uptake but this approach doesn’t allow to remove iron that has already accumulated in the body and also reduces the absorption of other elements contained in iron-rich foods, which are important to maintain a healthy status.

Bioactive molecules and dietary habits can affect iron status both directly by modulating its absorption during digestion and indirectly by epigenetic control of genes involved in its uptake, storage and recycling. Therefore, although further studies are required to deeply understand the role of the interaction between bioactive molecules and epigenetic modifications in triggering or preventing HH, nutritional interventions aimed at improving iron status may represent useful and safe strategies supporting phlebotomy in the management and treatment of HH, as well as other diseases that are related to the perturbation of iron homeostasis.

## Figures and Tables

**Table 1:** Dietary bioactive molecules and their impact on iron metabolism.

Molecules	Source	Action on Iron Absorption	Mechanism of Action	Evidence in HH Patients or Animal Models	Ref
**Vitamin C**	Mineral	Antioxidant/enhancer	Redox/chelation	Reduction of iron stores	[53]
**Phytates**	Plants	Inhibition	Chelation	Improvement of iron-induced oxidative stress and liver injury in animal models	[90]
**Polyphenols**	Plants	Inhibition	Chelation	Reduction of iron absorption and of the frequency of phlebotomies	[100,105]
**Calcium**	Mineral	Inhibition	Reducing iron uptake	Not investigated	
**Soybean, milk and egg proteins**	Animal	Inhibition	Unknown	Reduction of serum ferritin	[120]
**Vitamin A**	Mineral	Enhancer	Hepcidin-dependent upregulation of hepatic *Hamp* and duodenal *Fpn*	Vitamin A deficiency reported in HH	[125,126]
**Vitamin E**	Mineral	Antioxidant	Scavenge ROS protecting membranes from lipid peroxidation	Vitamin E deficiency reported in HH	[166,167,168,169,170,171,172,173,174]
**“Meat factor”**	Animal	Enhancer	Chelation	Not investigated	
**Orange/bergamot flavonoid-rich extracts**	Plants	Antioxidant	Chelation of iron and reduction of ROS	Not investigated	[175]
**EGCG**	Plants	Antioxidant/inhibitor	Chelation, reduction of basolateral iron export and activation of Nrf2 in Caco-2 and in human mesenchymal stem cells	Not investigated	[176,177,178]
**GSE**	Plants	Antioxidant/inhibitor	Chelation, reduction of basolateral iron export in Caco-2	Not investigated	[179]
**Curcumin**	Plants	Antioxidant	Reduction of iron content of liver, spleen and bone marrow; activation *TfR-1* and *IRP*, repression of hepatic ferritin and hepcidin synthesis	Not investigated	[180]
**Ferulic acid**	Plants	Antioxidant	Reduction of liver damage by increasing hepatic antioxidants and mitochondrial membrane potential	Not investigated	[181,182]
**Resveratrol**	Plants	Antioxidant	Upregulation of *SIRT1* expression and activation of FOXO1-dependent anti-oxidant response	Not investigated	[183,184]

HH: Hereditary hemochromatosis; *Fpn*: ferroportin; *Hamp*: Hepcidin antimicrobial peptide gene; ROS: Reactive oxygen species; Nrf2: Nuclear factor erythroid 2 related factor 2; TfR-1: Transferrin receptor 1; *IRP*: Iron regulated protein; *SIRT1*: Sirtuin 1; FOXO1: Forkhead box O1.

## Abbreviations

Dcytb	Duodenal Cytochrome b
DGCR8	DiGeorge Syndrome Critical Region 8
DIOS	Dysmetabolic Iron Overload Syndrome
DMT1	Divalent Metal Transporter 1
EGCG	Epigallocatechin-3-gallate
EPIC	European Prospective Investigation of Cancer
FOXO1	Forkhead Box O1
Fpn	Ferroportin
Glut1	Glucose Transporter 1
GSE	Grape Seed Extract
Hamp	Hepcidin Antimicrobial Peptide Gene
HCC	Hepatocellular Carcinoma
HFD	High Fat Diet
HH	Hereditary Hemochromatosis
HIF-1α	Hypoxia-Inducible Factor-1α
Hjv	Hemojuvelin
HSCs	Hepatic Stellate Cells
IR	Insulin Resistance
IRP1	Iron Regulated Protein-1
ITT	Insulin Tolerance Test
MetS	Metabolic Syndrome
miRNAs	microRNAs
NAFLD	Nonalcoholic Fatty Liver Disease
NHANES	National Health and Nutrition Examination Survey
Nrf2	Nuclear Factor Erythroid-2 Related Factor 2
ROS	Reactive Oxygen Species
SIRT1	Sirtuin 1
SNPs	Single Nucleotide Polymorphisms
Socs3	Cytokine Signaling-3
T2D	Type 2 Diabetes Mellitus
TfR-1	Transferrin Receptor-1
TNF-α	Tumor Necrosis Factor-alpha
VLDL	Very Low-Density Lipoprotein
